# Child feces management practices and fecal contamination: A cross-sectional study in rural Odisha, India

**DOI:** 10.1016/j.scitotenv.2019.136169

**Published:** 2020-03-20

**Authors:** Valerie Bauza, Fiona Majorin, Parimita Routray, Gloria D. Sclar, Bethany A. Caruso, Thomas Clasen

**Affiliations:** a Department of Environmental Health, Rollins School of Public Health, Emory University, Atlanta, GA, United States of America; b London School of Hygiene and Tropical Medicine, London, United Kingdom; c Independent Consultant, Bhubaneswar, Odisha, India

**Keywords:** Child feces, Fecal contamination, Sanitation, Hygiene, Open defecation

## Abstract

Safe child feces management (CFM) is likely critical for reducing exposure to fecal pathogens in and around the home, but the effectiveness of different CFM practices in reducing fecal contamination is not well understood. We conducted a cross-sectional study of households with children <6 years in rural Odisha, India, using household surveys (188 households), environmental sample analysis (373 samples for 80 child defecation events), and unstructured observation (33 households) to characterize practices and measure fecal contamination resulting from CFM-related practices, including defecation, feces handling and disposal, defecation area or tool cleaning, anal cleansing, and handwashing. For environmental sampling, we developed a sampling strategy that involved collecting samples at the time and place of child defecation to capture activity-level fecal contamination for CFM practices. Defecating on the floor or ground, which was practiced by 63.7% of children <6 years, was found to increase *E. coli* contamination on finished floors (*p* < 0.001) or earthen ground surfaces (*p* = 0.008) after feces were removed, even if paper was laid down prior to defecation. Use of unsafe tools (e.g., paper, plastic bag, straw/hay) to pick up child feces increased *E. coli* contamination on caregiver hands after feces handling (*p* < 0.0001), whereas the use of safe tools (e.g., potty, hoe, scoop) did not increase hand contamination. Points of contamination from cleaning CFM hardware and anal cleansing were also identified. The most common disposal location for feces of children <6 years was to throw feces into an open field (41.6%), with only 32.3% disposed in a latrine. Several households owned scoops or potties, but use was low and we identified shortcomings of these CFM tools and proposed alternative interventions that may be more effective. Overall, our results demonstrate the need for CFM interventions that move beyond focusing solely on feces disposal to address CFM as a holistic set of practices.

## Highlights

• Sampling at the time and place of child defecation measured fecal contamination from CFM practices.
• Current CFM practices were found to increase fecal contamination in the environment and on hands.
• CFM hardware of potties and scoops can reduce fecal contamination, but had low use and shortcomings.
• CFM interventions must address CFM as a holistic set of many practices beyond only feces disposal.

## Graphical abstract

**Figure:**
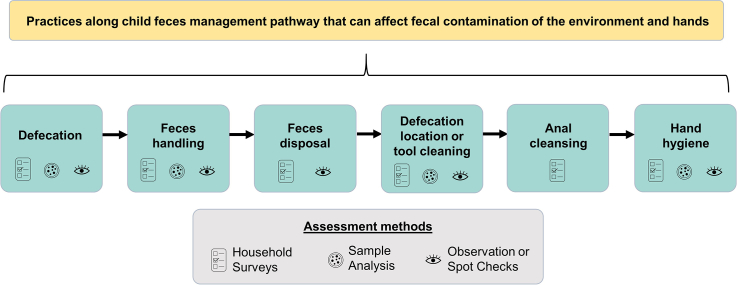

## 1 Introduction

Poor sanitation can lead to exposure to fecal pathogens and is associated with a heavy disease burden, including diarrheal disease, soil-transmitted helminth infections, trachoma, schistosomiasis, and nutritional deficiencies (Freeman et al., 2017; Prüss-Ustün et al., 2019). Fecal pathogens are transmitted via fecal-oral pathways including water, fields/soil, hands, flies, and food (Wagner and Lanoix, 1958). Improvements in household water quality and handwashing can reduce transmission along certain pathways, primarily water and hands. Adequate sanitation that contains human excreta should prevent fecal contamination of the environment and, subsequently, improve health. However, many recent sanitation intervention trials which have increased latrine access or quality have found no effect of sanitation on child diarrhea (Clasen et al., 2014; Humphrey et al., 2019; Null et al., 2018; Patil et al., 2014; Pickering et al., 2015) and/or stunting (Humphrey et al., 2019; Luby et al., 2018; Null et al., 2018). Consistent with these findings, the studies which also measured fecal contamination found that sanitation interventions did not reduce fecal indicator bacteria (i.e. *E. coli*) at potential household exposure points (Ercumen et al., 2018a, Ercumen et al., 2018b). This is consistent with a recent systematic review of the effect of sanitation interventions on fecal exposure, which found interventions resulted in little to no reduction in fecal contamination (measured by enteric pathogens or fecal indicator bacteria) along fecal-oral transmission pathways (Sclar et al., 2016). These results suggest that there are other sources of fecal contamination that are not adequately eliminated by typical sanitation interventions.

One potential remaining source of fecal contamination after sanitation interventions is poor child feces management (CFM). Sanitation interventions often focus on providing or improving a household's latrine/toilet facility or downstream fecal sludge management (WHO, 2018). In settings where open defecation is still common despite sanitation access, interventions may also focus on increasing latrine use. However, these interventions are largely targeted at adults and/or older children while comparatively little attention is paid to the safe collection and disposal of young child feces (Garn et al., 2017). Unsurprisingly, it cannot be expected that sanitation interventions designed to target latrine use among adults can succeed at altering the behavior of children who may be too young to use a latrine themselves, or may not be trained or encouraged to use a latrine. If children are not using a latrine directly, then additional work is required of the caregiver to safely pick up and dispose of children's feces into a latrine. As such, many household caregivers report that they do not dispose of their young children's feces in a latrine, despite having access to one (Bauza et al., 2019b; Bauza and Guest, 2017; Freeman et al., 2016; Majorin et al., 2017). Additionally, even when a CFM hardware intervention (i.e. an intervention of a physical infrastructure or product, such as a potty or scoop) is promoted alongside latrine improvements, there may still be substantially lower levels of safe sanitation practices for child feces compared to adults feces (Parvez et al., 2018).

Inadequate CFM practices present a health risk because the feces from children likely contain higher pathogen loads than feces from adults due to young children having poorly developed immune systems and higher incidence of enteric infections (Feachem et al., 1983; Walker et al., 2012). Young children are also at increased risk of exposure to other children's feces because children tend to defecate in areas near households where other young children may play or spend time outside (Lanata et al., 1998), and young children commonly engage in exploratory mouthing behaviors such as putting hands, objects, and soil into their mouths (Bauza et al., 2017, Bauza et al., 2018; Kwong et al., 2016; Moya et al., 2004; Ngure et al., 2013). Consistent with the increased risk of exposure, observational studies have found poor child feces disposal practices to be associated with diarrhea (Gil et al., 2004), soil-transmitted helminth infection (Roy et al., 2011), environment enteric dysfunction (George et al., 2016), and stunting (Bauza and Guest, 2017) in children.

While safe management of children's feces is likely critical for reducing fecal contamination exposure, the effectiveness of different CFM practices in reducing fecal contamination is not well understood. Past guidelines for CFM have often defined “safe” child feces disposal as a child using or their feces being disposed of into any toilet/latrine (Water and Sanitation Program, 2015), however it is now recognized that the toilet/latrine facility must be improved (i.e. a private flush/pour-flush toilet to a piped sewer system, septic tank, or pit latrine, a pit latrine with slab, ventilated improved pit latrine, or composting toilet) in order for disposal to be adequate and safe (WHO/UNICEF, 2018). Additionally, burial and disposal with solid waste have also sometimes been consider safe disposal methods, but an expert consultation deemed these methods unsafe (Bain and Luyendijk, 2015). However, other steps related to CFM are not currently included in these definitions of “safe” and “unsafe”, yet in addition to contamination introduced by an unsafe child feces disposal location, fecal contamination of the environment can also be caused by practices related to the defecation site, anal cleansing procedure, cleaning practices for tools used to assist with defecation or feces disposal, and hand contamination from handling feces (Majorin et al., 2017). Previous research has identified unsafe CFM as a potential source of fecal exposure in Odisha, India, but did not attempt to quantify the level of contamination associated with the practices (Majorin et al., 2014, Majorin et al., 2017). Understanding the extent to which these practices actually affect fecal contamination of the household environment could help determine the potential benefit, if any, of introducing CFM tools such as potties or scoops designed to collect and transport child feces to the latrine. It could also clarify which specific behaviors along the CFM chain of events might be more important to prioritize with interventions for fecal contamination reduction.

There are limited evidence-based recommendations for CFM in low-income settings in India and elsewhere. The objective of this study was to describe different practices for managing child feces at the household level and to determine how different practices are associated with contamination of caregiver hands and the environment. We used a combination of household survey, environmental sample analysis, and unstructured observation techniques in households residing in rural parts of Odisha, India.

## 2 Materials and methods

### 2.1 Study site

This study took place in rural villages in Puri district in Odisha, India, from September to October 2018. This study was supplemental to an ongoing 66 village cluster-randomized trial (CRT) evaluating a sanitation intervention to increase latrine use among all household members described in detail elsewhere (Clinicaltrials.gov: NCT03274245) (Caruso et al., 2019). In addition to promoting latrine use, the intervention included targeted CFM activities carried out in July 2018, approximately 2–4 months before this study. Activities were intended to instruct, motivate, and enable mothers of children under five years old to manage child feces safely and dispose feces in the latrine, and included the distribution of locally available potties and scoops (a plastic dustpan that could be used for CFM; shown in Fig. S1 in SI). Our study took place in six villages that were separate from the 66 CRT villages, but were purposively selected from the total villages eligible for the CRT (Caruso et al., 2019). Of these six villages, three received the intervention including the CFM hardware, and three were treated as control. The six villages were purposively selected to include one control and intervention village in each of three study blocks and to vary in size and latrine coverage similar to the variation in CRT villages. These six villages were separate from the CRT villages to allow for more in-depth engagement with households in qualitative data collection activities around latrine use and CFM practices without disrupting the actual trial. We included both intervention and control villages in this study to understand the potential for fecal contamination from a variety of CFM practices including the use of potties and scoops. However, our purpose is not to do an intervention vs. control comparison of CFM practices as the larger CRT is designed and powered for that comparison. Therefore, unless otherwise noted our results present data pooled from both intervention and control villages.

### 2.2 Study participants

All households with children under 6 years of age in the study villages were approached for inclusion in this study. Lists of households with children under 6 were provided by each village's Anganwadi center, which had records of the birthdates for village children. Each household from the lists were then visited by enumerators. Across the six villages, the lists included 232 households with potentially eligible children. However when households were visited, the child or entire household was absent for 41 households (typically because the child was currently staying at their uncle's or grandparent's house in a different village) and 3 households chose not to participate, with 188 households remaining that participated in our study. In these households, all children under 6 years were eligible to participate for the sample collection and unstructured observation activities and the children's primary caregiver (e.g., mother) was interviewed for the household survey. We included all children under 6 years in this study (instead of limiting it to children under 5 years like the CRT), because previous research in Odisha found that children were a median age of 5 years when a caregiver expected them to use a latrine on their own (Majorin et al., 2017) and we wanted to collect samples from children in the relevant age group that may be defecating in the household or compound.

### 2.3 Overview of data collection activities

**Fig. 1:**
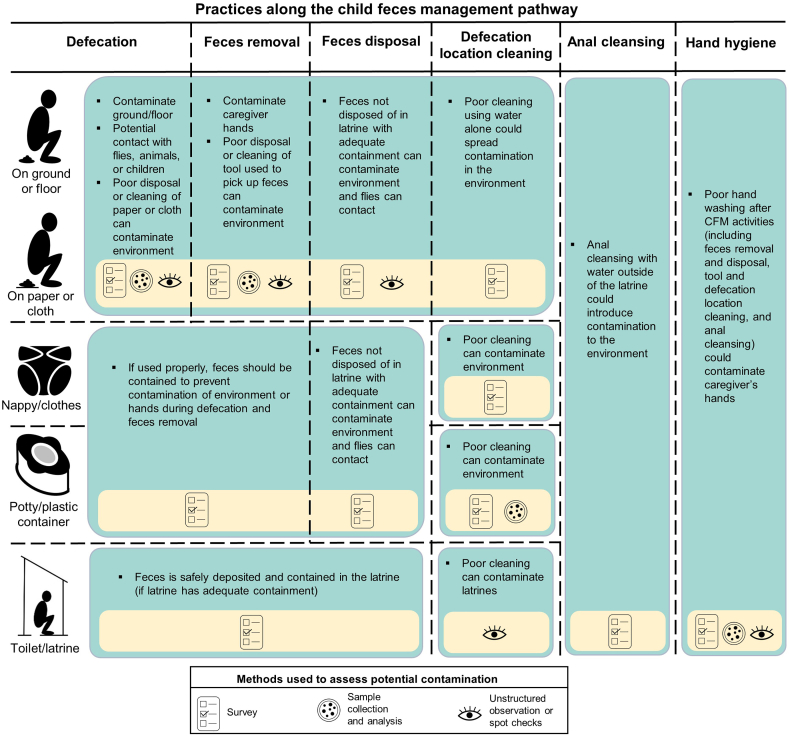
Potential sources where fecal contamination could enter the environment during practices along the CFM pathway and the methods used in this study to assess potential fecal contamination from each CFM practice.

Information about CFM management was collected using mixed methods, including household surveys, activity-level environmental sampling related to CFM practices after child defecation, and informal unstructured observation during sampling activities in a sub-set of households (Fig. 1). Household surveys were conducted in all 188 participating households is the six villages. Microbial sampling of activities related to CFM was conducted in a subset of households in five villages (three receiving the intervention and two control). As a child needed to defecate while enumerators were present for sampling to be completed, fewer households participated in sample collection (N = 73) than in household surveys. Sampling was only conducted in five of the six villages due to logistical constraints. Unstructured observation was performed in a subset of the households participating in microbial sampling (N = 33). Two enumerators from rural Puri conducted household surveys and sample collection activities after completion of a training session, and a separate observer conducted unstructured observations. The enumerators were also part of the field team for the larger CRT and had several years of experience working on previous sanitation studies in Odisha.

### 2.4 Household surveys

Enumerators interviewed the primary caregiver to collect information related to household demographics, water, sanitation and hygiene (WASH) infrastructure, sanitation practices of all household members, and CFM practices. For CFM specifically, information for all children under 10 years was collected related to their defecation location and who assisted the child with defecation (if anyone). If the place of defecation was someplace other than directly in a latrine, information was also collected on how long the feces remained at the place of defecation, how the feces were picked up, how the defecation area was cleaned, how materials used to pick up the feces, if any, were disposed of or cleaned, where the feces were disposed of, as well as how and where anal cleansing was performed. Caregivers' perceptions of potty and latrine use related to children's age were also captured during the household survey. If present, spot check observations of a household's latrine and designated place for handwashing were also conducted during the survey. Surveys were conducted in the local Odia language. The survey tool was developed based on questions from the baseline survey in the CRT villages that was previously piloted and completed as well as questions from other previous surveys in rural Odisha. Survey data was recorded on mobile phones using Open Data Kit software (available from https://opendatakit.org/).

### 2.5 Sample collection and processing

In order to characterize the levels of fecal exposure to which the child may be exposed due to various CFM practices, samples were collected from multiple points along the CFM pathway (Fig. 1). We designed our sampling strategy to collect samples at the place of child defecation and feces handling when these activities occurred to try to get more targeted activity-level evidence of contamination related to specific CFM activities. Our sampling strategy also involved measuring existing background contamination of *E. coli* in the environment or on hands to capture the level of contamination that was already present prior to child defecation or feces disposal activities.

As sampling needed to occur directly following child defecation before feces were disposed of, households were approached for their informed consent and to explain the sample collection activities at least one day before sampling was to occur. Households that agreed to participate were then reminded by enumerators about sampling in the morning on the day that sampling was to occur, and household members were asked to notify enumerators either when a child was ready to defecate or after the child had defecated before the feces were disposed of. Household members were informed of a nearby location that enumerators would be waiting in the village, and they either called enumerators or sent a household member to notify the enumerators in-person. Samples were only collected following morning defecation events due to the constraint that samples had to be transported to the lab and analyzed within the same day of sample collection. Samples were collected from 73 households and enumerators were present at each of these households so sample collection could begin before the feces were handled or disposed of by caregivers. The specific types of samples collected varied by household based on the location of defecation and the practices used for feces disposal. Regardless of the location of defecation, child feces samples, samples from the location of defecation, and caregiver hand samples were collected, with caregiver hand samples collected before and after any feces handling. We collected samples of actual feces in order to compare them directly with environmental samples.

If the child defecated directly on the floor or ground, a sample was collected from the floor or ground at the place of defecation after the feces were removed. If the child instead defecated on a material that was laid down on the floor or ground, a sample was collected from the floor or ground at the place of defecation after this material and the feces were removed. In order to use the samples collected at the place of defecation to estimate the level of fecal contamination added to the floor or ground from child defecation practices, we also sampled a place approximately 30 cm away from the place of defecation to estimate the existing baseline level of contamination of the floor or ground.

Samples were also collected from potties and scoops. If a child defecated in a potty, a wash water sample was collected from the potty bowl after the caregiver cleaned the potty in her typical manner. If a scoop was used, a swab sample of the scoop was also taken after the caregiver cleaned the scoop in her typical manner. No instructions related to the method of cleaning the potty or scoop were given to the caregiver, however, the typical manner of cleaning commonly involved the use of water or water and soap. These samples were intended to assess the contamination that remained after cleaning to provide an estimate of the lower limit of potential contamination of the environment from wash water if either item is washed in an open location, such as at a handpump or in surface water. These samples also provided an estimate of potential exposure if a child was to come in contact with the potty or scoop surface where it was expected to have the highest level of contamination.

#### 2.5.1 Fecal samples

Caregivers were instructed to use a sterile polystyrene sampling spoon (Nasco, Fort Atkinson, WI, USA) to collect a sample of a child's feces from the place of defecation before the caregiver picked up the feces to dispose of it. The caregiver was instructed to do this in a gentle manner coming from underneath the feces so as not to increase the contamination of the soil or surface underneath the feces during sampling.

#### 2.5.2 Surface soil samples

To mark the location of the child's feces on the ground before it was removed, toothpicks were placed on all four sides of the feces. If a material (such as paper) was laid down prior to defecation, then toothpicks were placed on all four sides of that defecation material before it was removed. After the caretaker removed feces (or the defecation material and feces, if applicable) from the ground following their normal practice, soil samples were collected by an enumerator from the surface at the place of defecation. Soil was also collected from a location approximately 30 cm away from the place of defecation. Soil was collected from this nearby location to serve as a measure of the existing soil contamination to compare with the soil contamination at the place of defecation, to estimate the increase in soil contamination following child defecation. To sample the soil at each location, a sterile polystyrene sampling spoon was used to collect approximately 10 g of surface soil from an area of approximately 10 cm by 10 cm, following the methods of Bauza et al. (2017).

#### 2.5.3 Finished floor samples

To mark the area where the child defecated on the floor (concrete or stone) or a material laid down on the floor, we followed a similar method using toothpicks as noted above. We collected samples from the place of defecation and a location approximately 30 cm away from the place of defecation, but used sterile nylon-flocked swabs stored in liquid Amies elution solution (BD ESwabTM, Franklin Lakes, NJ) for sampling the floor surface. Swab sample collection and analysis followed methods modified from Hedin et al. (2010). Swabs were removed from the elution solution, pressed against the side of the tube to remove excess solution from the swab, and then the tip was used to swab an approximately 10 cm by 10 cm square of the floor, thoroughly wiping in the horizontal direction, and then the vertical direction of the square, constantly rotating the swab head while swabbing the surface. The swab was returned to the tube and stored in the elution solution until processing.

#### 2.5.4 Scoop samples

Scoops were sampled after the caregiver used it to pick up the child feces and cleaned it following her usual practice. The swabbing protocol followed the steps outlined above for floor samples, except the entire scoop's top surface (excluding the handle) was sampled instead of a 10 by 10 cm area.

#### 2.5.5 Potty samples

Potties were sampled after the caregiver disposed of the feces and cleaned it following her usual manner. To sample potties, 200 ml of sterile phosphate buffer solution (PBS) was poured into the potty bowl and a sterile sampling spoon was used to mix the PBS for 30 s within the potty. The PBS was then poured into a sterile whirl-pak bag for collection.

#### 2.5.6 Hand rinse samples

Two hand rinse samples were collected from each caregiver: the first was collected prior to picking up or handling their child's feces and the second was collected after picking up their child's feces to dispose of it. Prior to taking the first hand sample, caregivers were instructed to clean their hands with waterless hand sanitizer following the methods of Pickering et al. (2010). Specifically, 2 ml of waterless hand sanitizer containing 70% ethyl alcohol (Purell brand, Gojo Industries, Akron, OH) was placed in one palm, and the caregiver was instructed to rub her hands together, following the procedure recommended by the WHO for hand hygiene technique using alcohol-based hand sanitizer (WHO, 2009), while an enumerator demonstrated the technique using her own hands. The first hand rinse sample was taken immediately after the hand sanitizer dried and the second hand rinse sample was taken after the caregiver returned to the house after disposing of the child feces. Caregivers were asked not to wash their hands after disposing of the feces prior to the hand sample to enable a comparison of relative hand contamination among different feces handling practices and because caregivers may not always wash their hands after handling their children's feces, especially if they are busy. Samples were collected by having the caregiver place each hand, one at a time, into a sterile whirlpak bag (Nasco) pre-filled with 200 ml of sterile PBS, for 20 s at a time while the enumerator massaged the hand through the plastic bag.

### 2.6 *E. coli* enumeration

*E. coli* were enumerated on sterile 47 mm diameter, 0.45 μm pore size filters composed of mixed esters of cellulose (MilliporeSigma, Burlington, MA) using m-ColiBlue24 Broth media (Hach, Loveland, CO). The manufacturer's protocol for membrane filtration method 10029 approved by the United States Environmental Protection Agency was followed (USEPA, 1999). Media plates with the filters were incubated at 35 °C for 24 h. Multiple dilutions of each sample were made using PBS and two or three dilutions of each sample were processed, depending on sample type. For soil samples, bacteria were eluted from soil into PBS prior to dilution, following the method described elsewhere (Bauza et al., 2017). For swab samples, the swab tube was vortexed for 20 s to elute the bacteria and then the swab head was pressed against the side of the tube walls to release liquid remaining on the swab head before processing and diluting the elution solution. All samples were stored in a cooler with ice packs after sample collection until they were processed in the lab within 12 h of collection. Sample pairs from the same household (e.g., caregiver hand samples before and after feces disposal or swab samples at and near location of defecation) were always processed at the same time in the lab to prevent differences in storage time before analysis. Lab and field blanks of the PBS used for sample collection and dilution were processed as negative controls: lab blanks were processed daily, and field blanks were processed by each enumerator once per week.

### 2.7 Unstructured observation

Unstructured observations related to child defecation, feces handling, and/or feces disposal were completed in a subset of 33 households during sample collection activities. Unstructured observation is a method of formative research that can provide insights into the context and process of activities, capture the whole picture, and illustrate the influence of the physical environment (Mulhall, 2003), and we used this technique in our study to gain additional context of CFM practices that may not have been readily captured from survey questions or sample collection. The unstructured observation period was defined as the period of time that the field enumeration team was in a household for sample collection activities, which was during the morning and lasted approximately 20 to 30 min per household. Unstructured observation was informal in nature, with characteristics of defecation (e.g., details of location and materials used for defecation), feces handling (e.g., details of the methods used by caregivers to pick up or remove feces), and feces disposal (e.g., details of feces disposal location) watched by a separate observer while enumerators completed sample collection activities in that household. Free-form written descriptive notes were recorded immediately following the observation period.

### 2.8 Statistical analysis

*E. coli* colony forming unit (CFU) counts were log transformed prior to analysis. For samples with *E. coli* CFU that were too numerous to count at the highest dilution of a sample (>200 CFU per filter), the upper limit of quantification (200 CFU at the highest dilution) was assigned to the sample. If the lowest dilution of a sample did not detect any *E. coli*, then half the limit of detection (LOD) was assigned to the sample. Differences in levels of contamination at the point of defecation compared to nearby point of sample collections for soil and floor samples were analyzed using paired *t*-tests. Paired *t*-tests were also used to test for differences in levels of contamination between hand samples taken before and after child feces handling. Stata 15.1 (StataCorp LLC, College Station, Texas, USA) was used for all analyses.

### 2.9 Ethical approval

Verbal informed consent was obtained from the primary caregiver prior to participation in study activities. This study was approved by the Institutional Review Board of Emory University (Ref. IRB00098293) and the Independent Ethics Committee of Xavier University in Bhubaneswar, Odisha, India (Ref. No. 131216).

## 3 Results

Information on defecation practices was collected for 1072 households members through household surveys, including detailed CFM practice information for 310 children under 10 years of age. In total, 373 samples were collected and analyzed for *E. coli*, including samples associated with the 80 children. As a child needed to defecate while enumerators were present for sampling to be completed, fewer households participated in sample collection (N = 73) than in household surveys (N = 188); all households that participated in sample collection also participated in the household survey. Six households were sampled multiple times, with samples collected for a different child in the household each time (i.e. five households had samples collected for two children and one household had samples collected for three children).

### 3.1 Household characteristics and defecation practices

Households included in our study had moderate levels of access to WASH infrastructure: 72.9% of households reported having access to a latrine, 73.4% reported having access to drinking water in their own dwelling or yard/compound, 34.0% reported access to an enclosed bathing area, and 19.2% reported access to a designated handwashing place. Over 97% of latrines were flush or pour-flush to a septic system or pit latrine and pictures of variations of latrines are provided in Fig. S2 (since latrine design is relevant for child latrine use). The majority of participants had received some level of formal education, with 83.5% of primary female caregivers and 87.7% of the male head of households reported to have completed primary education or higher. As a proxy of household wealth, 96.3% of households had electricity, 95.7% had a phone, 64.9% owned a mattress, 54.3% owned a motorcycle/scooter, and 47.9% owned livestock. All households reported being of Hindu religion.

Nearly all household members (95.4%) were reported by the survey respondent to defecate either one or two times per day (Table 1). Most (60.8%) were reported to have defecated in a latrine the last time they defecated, including 71.5% of household members between the ages of 15–59 years (Table 1). Analyzing by sex, women aged 15–59 years were more likely to defecate in a latrine than men (reported for 78.3% of women and 64.0% of men) and men in this age group were more likely to defecate on the ground outside the compound (i.e. practice open defecation) than women (reported for 18.8% of women and 27.7% of men). There were minimal differences in latrine usage or defecation locations by sex for other age groups (Table S1, Supplemental Information).

**Table 1:** Defecation practices reported for each household member, separated by age category. The results shown are for all included households from study villages in Odisha, India.

N	<3 years	3–5 years	6–9 years	10–14 years	15–59 years	60+ years
	102	124	84	36	586	140
*Defecation frequency* (*times/day*)						
1	44.1%	46.0%	51.2%	44.4%	38.7%	26.4%
2	38.2%	45.2%	39.3%	52.8%	59.6%	71.4%
3	11.8%	7.3%	4.8%	2.8%	1.4%	0.7%
4 or more	5.9%	0.8%	3.6%	–	0.2%	1.4%
Other: <1 time/day	–	0.8%	1.2%	–	–	–

*Defecation location* (*the last time this person defecated*)						
In latrine	3.9%	37.1%	70.2%	80.6%	71.5%	67.9%
On ground outside compound	5.9%	11.3%	20.2%	13.9%	23.0%	30.7%
On ground inside compound	29.4%	40.3%	9.5%	2.8%	0.3%	–
On floor in house	37.3%	4.8%	–	–	–	–
In potty	5.9%	2.4%	–	–	0.2%	–
In cloth nappy/diaper	2.0%	–	–	–	–	–
In pants/clothing	2.9%	0.8%	–	–	–	–
On bed	9.8%	–	–	–	–	–
In bedpan	–	–	–	–	0.2%	0.7%
Other	–	1.6%	–	–	–	–
Don't know	2.9%	1.6%	–	2.8%	4.8%	0.7%

*Other common defecation locations*^a^						
In latrine	–	3.3%	1.2%	–	2.2%	2.9%
On ground outside compound	1.0%	1.6%	4.8%	2.8%	5.0%	7.9%
On ground inside compound	8.8%	11.4%	2.4%	–	0.2%	0.7%
On floor in house	10.8%	8.1%	–	–	–	–
In potty	1.0%	–	–	–	–	–
In cloth nappy/diaper	1.0%	–	–	–	–	–
In pants/clothing	8.8%	–	–	–	–	–
On bed	3.9%	–	–	–	–	–
In bedpan	–	–	–	–	–	–
Don't know	2.9%	1.6%	–	2.8%	5.0%	1.4%
No other place of defecation	62.8%	75.6%	91.7%	94.4%	87.7%	87.1%

a Summarizes responses to the survey question “Are there any other places this person commonly defecates?” which was asked after asking the location of defecation for the last time this household member defecated. Respondents were allowed to select as many additional locations as applicable.

### 3.2 Caregiver-reported CFM practices

#### 3.2.1 Defecation

Child defecation practices varied by child's age, with younger children (<3 years) being most likely to defecate on the floor inside the household (37.3%) or on the ground inside the household compound (29.4%), children aged 3–5 years being most likely to defecate on the ground inside the household compound (40.3%) or in the latrine (37.1%), and children aged 6–9 years being most likely to defecate in the latrine (70.2%; Table 1).

As expected, a child's ability to walk or not influenced their defecation site. The majority of children (84.4% of 32) that were not yet able to walk without assistance (i.e. “pre-ambulatory”) were <1 year old. Only pre-ambulatory children were reported to defecate in a cloth nappy/diaper, in pants/clothing, or on the bed — except for one ambulatory child that reported defecating on the bed. Overall, 6.3% of pre-ambulatory children were reported to defecate in a cloth nappy/diaper, 12.5% in pants/clothing, and 28.1% on the bed. However, it was also common for pre-ambulatory children to defecate on the ground inside the compound (18.8%) or on the floor inside the household (34.4%). Additionally, when asked if there were any other places each household member commonly defecates, caregivers responded that many younger children had at least one other common place of defecation (reported for 37.2% of children <3 and 24.4% of children 3–5 years) but few older children did (reported for only 8.3% of children 6–9 years; Table 1).

When children defecated on the floor or ground, it was most common (62.7%) for them to do so directly, with no material placed down before they defecated, regardless of the child's age (Table 2). However, when a material was placed on the floor or ground, it was usually waste newspaper/paper (35.5%; Table 2).

**Table 2:** Child feces management (CFM) practices related to defecation materials laid down, defecation area cleaning, feces handling, and feces disposal. The results shown are for all included households from study villages in Odisha, India.

Total N	<3 years	3–5 years	6–9 years	All <10 years
	102	124	84	310
*Defecation material laid down* (*if defecated on ground or floor*)				
N	74	70	25	169
Waste newspaper/paper	39.2%	37.1%	20%	35.5%
Cloth	2.7%	–	–	1.2%
Polythene/plastic bag	–	1.4%	–	0.6%
Nothing	58.1%	61.4%	80%	62.7%

*Defecation area cleaning* (*if defecated on ground or floor*)				
N	74	64	10	148
With water	77.0%	70.3%	50%	72.3%
With soap and water	1.4%	–	–	0.7%
With disinfectant and water	13.5%	3.1%	10%	8.8%
With cow dung	4.1%	3.1%	–	3.4%
With cow dung and water	1.4%	10.9%	10%	6.1%
With cloth	2.7%	–	–	1.4%
Not cleaned	–	12.5%	30%	7.4%

*Material used to pick up feces* (*if picked up*)				
N	74	64	10	148
Waste newspaper/paper	74.3%	67.2%	60.0%	70.3%
Cloth	8.1%	–	–	4.1%
Polythene/plastic bag	5.4%	1.6%	–	3.4%
Leaf	1.4%	–	–	0.7%
Straw/hay	8.1%	25.0%	20.0%	16.2%
Scoop	2.7%	1.6%	–	2.0%
Hoe	–	4.7%	20.0%	3.4%

*Feces disposal location*				
N	102	124	84	310
Child used latrine	3.9%	36.3%	70.2%	34.8%
Put/rinsed in latrine	16.7%	5.7%	–	7.7%
Put/rinsed in drain/ditch	1.0%	1.6%	–	1.0%
Thrown in garbage	11.8%	8.1%	2.4%	7.7%
Thrown in open field	43.1%	40.3%	9.5%	32.9%
Left in open	–	5.7%	17.9%	7.1%
Put/rinsed in pond/surface water	14.7%	0.8%	–	5.2%
Washed away with soap and water	5.9%	–	–	1.9%
Don't know	2.9%	1.6%	–	1.6%

Although 32.5% of households reported owning a plastic potty, the majority of which were distributed to households as part of the ongoing intervention study, only 5.9% of children <3 years and 2.4% of children 3–5 years were reported to defecate into the potty (Table 1). Among respondents who had heard of potties (43.1% of all respondents, 67.7% of respondents in villages that received the intervention), the median age reported for when they thought it was appropriate for a child to begin using a potty was 1 year (interquartile range [IQR] 1–2, range 0–4; Table S2 in SI) and the median age that they reported a child being too old to use a potty was 3 years (IQR 3–5, range 1–6; Table S2).

For child latrine use, respondents reported a median age of 4 years (IQR 3–5, range 1–7; Table S2) for when they did or will begin training their child to use a latrine and a median age of 6 years (IQR 5–8, range 3–10; Table S2) for when they thought that a child should be able to use a latrine on their own without assistance. When asked if there were any reasons that a child who is 3 or 4 years old should not use a latrine to defecate, 61.7% of respondents (116 out of 188) reported that there was no reason a child should not use a latrine at this age. Fear and safety were the most common answers selected as reasons a child aged 3 or 4 years should not use the latrine: 28.2% of respondents reported that a child could fall into the squatting pan or pit and 13.3% reported a child may be scared to use the latrine. Alternatively, few respondents (3 respondents; 1.6%) reported a child would dirty the latrine as a potential reason not to use the latrine, indicating this is likely not a common factor limiting child latrine use in this area. Only 1 respondent reported that it takes too much time to teach a child to use the latrine and she also reported that a child this age (3 or 4 years) should use a latrine (as respondents were allowed to have more than one reason), indicating that they did not view the training time as a valid reason for a child not to use a latrine.

#### 3.2.2 Feces handling and removal

When caregivers picked up children's feces to dispose of it, waste newspaper/paper was most commonly used (70.3%; Table 2). Although 47.9% of households reported owning a scoop (the majority of which were distributed to households as part of the intervention study), only 2.7% of children <3 years and 1.6% of children 3–5 years were reported to have their feces picked up using a scoop (Table 2).

#### 3.2.3 Feces disposal

Feces disposal locations also varied by child's age, with feces being thrown into an open field being the most common feces disposal location for children <3 years (43.1% for all households, 43.6% for households with a latrine) and 3–5 years (40.3% for all households, 27.4% for households with a latrine) and a child using the latrine being the most common feces disposal location for children 6–9 years (70.2% for all households, 85.7% for households with a latrine; Table 2). Safe disposal of child feces into a latrine was rare for children <3 years (20.6% for all households, 25.6% for households with a latrine) and insufficient for those 3–5 years (42.0% for all households, 50.8% for households with a latrine; Table 2). 82.6% of children <10 years with feces disposed of in the latrine (109 out of 132) were reported to defecate directly in the latrine, demonstrating that few households dispose of children's feces into a latrine when it is picked up from other defecation locations. In fact, when excluding children who defecated in the latrine directly, only 18.2% of children's feces among households with a latrine (or 11.7% for all households) was picked up and disposed of in the latrine by caregivers. Unlike the defecation site, it was rare for child feces to be disposed of in multiple locations. Instead, for 91.3% of children <10 years, there was only one common disposal location reported.

#### 3.2.4 Defecation area or tool cleaning

When a child defecated on the ground or floor and the feces was removed, the defecation areas for children under 10 years were commonly cleaned using only water (72.3%; Table 2), which has the potential to spread the fecal contamination left on the ground, rather than eliminating this contamination.

Additionally, when potties and soiled clothes/nappies were cleaned, the wash water from cleaning them was often disposed of in an unsafe way that could contaminate the environment, though this was more common for wash water from soiled clothes/nappies than for potties. Of children who used a potty, the majority (77.8%, N = 7) of potties were reported to be washed in the latrine with wash water also disposed of in the latrine. The remaining potties were either washed outside the house in or near the compound with wash water disposed of on the garbage pile (11.1%, N = 1, household had a latrine) or cleaned at a pond/surface water with wash water disposed of in that surface water (11.1%, N = 1, household did not have a latrine). Of children who ever defecated in clothes or cloth nappies, only about one-fifth of these soiled clothes/nappies (21.4%, N = 3) were reported to be washed in the latrine with wash water also disposed of in the latrine. The remaining soiled clothes/nappies were either cleaned at a pond/surface water (35.7%, N = 5) with wash water disposed of in that surface water (28.6%, N = 4, 2 of the households had a latrine) or onto the garbage pile (7.1%, N = 1, household had a latrine), washed at the handpump with wash water disposed of near the handpump (21.4%, N = 3, all households had latrine), or washed outside the house in or near the compound with wash water disposed of on the ground outside the house (21.4%, N = 3, 1 household had latrine).

Only one caregiver that reported using the scoop to pick up their child's feces also reported washing the scoop with water after use. For this household, it was reported to have been washed outside the house in or near the compound, which has potential to introduce fecal contamination into the environment.

#### 3.2.5 Anal cleansing

Anal cleansing of children was commonly practiced using water, which was reported for 78.4% of children <3 years, 96.8% of children 3–5 years, and 94.1% of children 6–9 years (Table 3). However, the location of anal cleansing may have the potential to introduce contamination into the domestic environment, as anal cleansing of children <3 years was reported to commonly occur at the handpump (50%; Table 3) and sometimes occur on the household floor (17.7%), on household furniture or bed (4.9%), or on the ground in the compound (13.7%). Similar locations were also common for children aged 3–5 years, whereas anal cleansing of children 6–9 years was commonly performed in the latrine (Table 3). Most children did not have another common location where anal cleansing was performed; no other location was reported for 89.3% of children <10 years.

**Table 3:** Child anal cleansing practices and locations. The results shown are for all included households from study villages in Odisha, India.

N	<3 years	3–5 years	6–9 years	All <10 years
	102	124	84	310
*Anal cleansing material used*				
Water	78.4%	96.8%	94.1%	90.0%
Soap and water	2.0%	1.6%	2.4%	1.9%
Wet wipes	15.7%	–	–	5.2%
Other	1.0%	–	–	0.3%
Don't know	2.9%	1.6%	2.4%	2.3%
Not cleaned	–	–	1.2%	0.3%

*Anal cleansing location*				
Inside household on floor	17.7%	5.7%	–	8.1%
Inside household on furniture/bed	4.9%	–	–	1.6%
Inside household in bucket	1.0%	–	–	0.3%
In compound on ground	13.7%	16.9%	3.6%	12.4%
In latrine over pan	2.0%	5.7%	30.1%	11.1%
In latrine not over pan	3.9%	16.1%	32.5%	16.6%
At handpump	50%	49.2%	20.5%	42.0%
In pond/surface water	–	4.0%	8.4%	3.9%
Other	3.9%	0.8%	3.6%	2.0%
Don't know	2.9%	1.6%	1.2%	2.0%

#### 3.2.6 Handwashing

Handwashing was frequently reported following CFM activities: 99.5% of caregivers reported always washing their hands after picking up their child's feces with 96.3% using soap and water. Additionally, 100% of caregivers reported washing their hands after cleaning their child post-defecation with 95.2% using soap and water. These results were similar to reported handwashing after personal toilet use; 91.5% of caregivers reported always washing their hands after toilet use and 4.8% reported sometimes washing their hands, with 95.2% reporting using soap and water at this time.

#### 3.2.7 Combined CFM pathway

It was uncommon for all practices for the same child along the CFM pathway to be carried out by the caregiver or child in a way that would eliminate the potential for fecal contamination to be introduced to the environment and be considered safe. Of the 305 children with full information collected for defecation, feces handling and disposal, CFM tool cleaning, and anal cleansing practices, the caregiver only reported that all of these CFM practices were safe for 34 children (11.2%) and all of these children used the latrine directly. This is less than the total number of children to defecate in the latrine because anal cleansing practices that used water were only considered safe if they were performed in the latrine over the pan. For children with safe defecation locations (e.g., contained locations such as latrine, potty, or reusable cloth diaper/clothes) and a safe disposal location into a latrine, practices related to anal cleansing using water in a location that could introduce contamination to the environment, such as in the latrine not over the pan or at the handpump, often resulted in an unsafe practice related to CFM for that child. This demonstrates the importance of capturing the full range of behaviors for CFM.

#### 3.2.8 Intervention vs. control comparison

Intervention and control villages had similar results related to potential CFM practices leading to contamination of the environment or hands and therefore results from all villages have been pooled together throughout the results. However, there was a higher level of latrine use (Table S3) and safe disposal into a latrine (Table S4) in intervention villages compared to control villages. Despite these increases, there were still many children reported to defecate on the floor or ground inside the household or compound and many children's feces that were disposed of into open fields or other unsafe ways in intervention villages. Additionally, practices that could lead to contamination along other steps of the CFM pathway were largely similar among intervention and control villages.

### 3.3 Fecal contamination measurements related to CFM practices

Overall, household visits resulted in 67 fecal samples, 43 pairs of samples from finished floor (concrete or stone in house or compound), 33 pairs from soil (earth ground), 73 pairs from caregivers' hands, 5 scoop samples, and 3 potty samples being collected and analyzed. Multiple samples had levels of contamination that were below the LOD for *E. coli*, including 9 fecal samples, 2 floor samples at the place of defecation, 7 floor samples at a place nearby defecation, 1 soil sample pair at both locations, 20 hand samples before picking up feces, 7 hand samples after picking up feces, and 1 potty sample. Additionally, multiple samples had levels of contamination that were above the upper limit of quantification (ULOQ), including 11 floor samples at the place of defecation, 2 floor samples at a place nearby defecation, 4 soil samples at the place of defecation, 1 soil sample at a place nearby defecation, 2 hand samples before picking up feces, 9 hand samples after picking up feces, and 1 scoop sample. All lab and field blanks processed as negative controls were negative for *E. coli*.

#### 3.3.1 Defecation

The contamination level of *E. coli* on household floors (in an approximate 100 cm^2^ area) increased by one order of magnitude (difference = 0.95 log CFU *E. coli*, *p* < 0.0001) and the contamination level of soil (in 1 g of soil) increased by approximately half an order of magnitude (difference = 0.47 log CFU *E. coli*, *p* = 0.008) after children defecated on the floor/soil directly or on a paper/cloth laid down and feces was removed (Table 4). For household floors, contamination increases were also observed individually whether no material was laid down (difference = 1.19 log CFU *E. coli*, *p* = 0.0002) or if waste paper/newspaper was laid down before defecation (difference = 0.82 log CFU *E. coli*, *p* = 0.0006). For soil, however, contamination increases were observed when no material was laid down before defecation (difference = 0.85 log CFU *E. coli*, *p* = 0.03), but not if waste paper/newspaper was laid down first (difference = 0.33 log CFU *E. coli*, *p* = 0.06).

**Table 4:** Differences in *E. coli* contamination in samples collected from finished floor (concrete or stone in household or compound) and soil (earth ground) sample pairs taken after child feces had been removed at the location of defecation and at a nearby location. Results are shown for different materials that were laid down prior to child defecation.

Material laid down before defecation	N	log (CFU *E. coli*) per 100 cm^2^		Paired *t*-test	
		At spot of defecation	Near spot of defecation	log *E. coli* difference	*p*-value
*Finished floor samples*					
No material	15	4.56	3.37	1.19	0.0002⁎
Waste newspaper/paper	27	3.83	3.02	0.82	0.0006⁎
Cloth	1	2.70	1.70	1.0	–
All samples	43	4.06	3.11	0.95	<0.0001⁎

*Soil samples*					
No material	9	4.56	3.71	0.85	0.03⁎
Waste newspaper/paper	24	3.77	3.44	0.33	0.06
All samples	33	3.98	3.51	0.47	0.008⁎

⁎ Significant result (*p* < .05).

#### 3.3.2 Feces handling and removal

The *E. coli* contamination level on caregivers hands after picking up and disposing of their children's feces was no different than the level of contamination before feces disposal when practices predicted to be safe practices (e.g. potties, hoes, scoops) were used (difference = −0.24 log CFU E. coli, *p* = 0.54; Table 5). However, when practices predicted to be unsafe (e.g. paper, straw/hay, plastic bag, cloth) were used to pick up child feces, the *E. coli* contamination level on hands increased (difference = 0.70 log CFU *E. coli*, *p* < 0.0001), with the highest increase seen when straw/hay was used to pick up feces, which increased contamination by more than two orders of magnitude (difference = 2.1 log CFU *E. coli*, *p* = 0.001; Table 5). Specific practices were pre-classified as safe or unsafe for this analysis based on reusability of tool and the expected barrier between hands and feces created by the material. Our results provide experimental evidence that supports our classifications of specific items as safe or unsafe. Cloth was one exception, with inconclusive evidence about whether it should be classified as a safe or unsafe material for feces handling. Cloth is reusable and we did not measure an increase in hand contamination when this tool was used, however we have conservatively classified it as unsafe due to the small sample size and large potential for variation in the type of cloth used from one household to the next.

**Table 5:** Differences in *E. coli* contamination in samples collected from caregiver hand pairs taken before and after a caregiver handles feces to dispose of it. Results are shown for different materials that were used to handle the child feces.

Material used to pick up feces	N	log (CFU *E. coli*) per 2 hands		Paired *t*-test	
		Before	After	log *E. coli* difference	*p*-value
Potty	3	2.94	2.66	−0.28	0.22
Scoop	3	1.75	1.50	−0.25	0.60
Hoe	2	2.95	2.78	−0.17	0.94
Waste newspaper/paper	53	2.09	2.68	0.60	0.0003⁎
Straw/hay	4	2.75	4.87	2.12	0.001⁎
Plastic bag	3	3.73	4.10	0.37	0.53
Cloth	3	1	1	–	–
Plastic bag & waste newspaper/paper	1	2.26	3.69	1.43	–
Scoop & waste newspaper/paper	1	2.11	4.60	2.49	–

Safe practices (potties, scoops, hoes)	8	2.50	2.26	−0.24	0.54
Unsafe practices (paper, straw/hay, plastic bag, cloth)	65	2.16	2.85	0.70	<0.0001⁎

⁎ Significant result (*p* < .05).

#### 3.3.3 Defecation area or tool cleaning

**Fig. 2:**
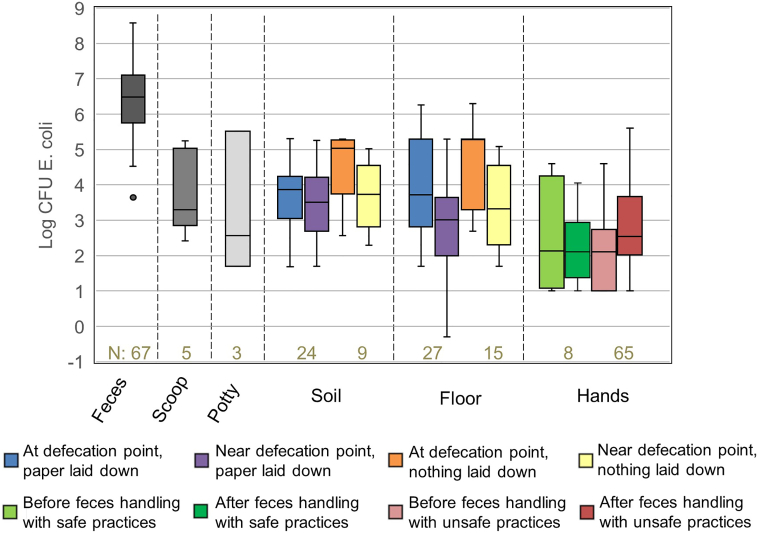
Level of *E. coli* contamination measured at different sampling locations. Log CFU *E. coli* is shown as per gram for feces and soil, per approximate 100 cm^2^ area for floor, and per scoop, potty, or two hands.

Scoops and potties remained contaminated with *E. coli* after they were cleaned by the caregiver following her usual practice (log mean of 3.8 CFU *E. coli* for scoops and 3.3 CFU *E. coli* for potties; Fig. 2). Although these measurements do not provide an precise estimate of the fecal contamination likely to enter the environment from unsafe cleaning/wash water disposal practices, they do provide an estimate of the lower limit of potential fecal contamination from wash water if either item is washed in an open location as well as an estimate of potential exposure if a child was to come in contact with the potty or scoop surface.

#### 3.3.4 Contamination variability

The levels of *E. coli* contamination measured varied considerably for the same locations among different households, even when the same materials were laid down on the floor or ground prior to defecation or the same materials were used by caregivers to pick up feces (Fig. 2). Specifically, *E. coli* counts in fecal samples varied more than five orders of magnitude, scoop samples varied almost three orders of magnitude, potty, soil, and hand samples varied about four orders of magnitude, and floor samples varied about five orders of magnitude.

### 3.4 Unstructured observations of child defecation and feces disposal events

#### 3.4.1 Defecation

Observations provided insights as to where children defecated in and around the household. It was common for defecation to occur in areas that appeared to be hallways or walkways, or other locations that were either inside or near (within a few feet of) the household, and/or near handpumps or latrines. Whether children defecate in a designated area or many different areas may vary by household. In some households, children appeared to defecate in a variety of locations rather one designated area, which may create potential for more dispersed contamination. Some children defecated onto different locations on the floor/ground within the house or compound, which became evident when we returned to the same household on another day to collect samples for another child. However, we also observed that children from different neighboring households sometimes defecated in similar places within the compound on different days, suggesting that some households may also have designated defecation areas on the ground that children frequently use.

In addition, we observed how caregivers engaged with their children during defecation events and how the behaviors and practices of children and caregivers varied. Caregivers commonly had their children squat for several minutes, including after an initial defecation event was completed, to ensure the child had finished. Sometimes the mother was closely watching the child while at other times the mother was doing other things when the child was squatting such as cooking nearby. When waste newspaper/paper was laid down by caregivers prior to a child defecating, it was common for the type, quantity, and thickness of paper to vary from household to household, ranging from a single sheet of newspaper/paper to multiple sheets of either. Sometimes a child also urinated while defecating, which left a wet spot on the paper below the feces. Finally, children in multiple households were observed to defecate on the floor/ground or paper laid down even though there was a child potty accessible in that household. In one household, a child was observed that appeared to enjoy sitting on and playing on a potty. However, the child appeared to be unable to defecate in the potty when the caregiver asked him to, but he was able to quickly defecate on the ground after the caregiver laid down a piece of paper for him to defecate on.

#### 3.4.2 Feces handling and removal

Observation of caregivers using the scoops revealed that other disposable items often needed to be used to push the feces onto the scoop, and these items could create additional feces-contaminated garbage. Specifically, caregivers often used a small wooden stick to push hard feces or paper to push soft feces from the ground onto the scoop. When children tried to defecate directly onto the scoop, the scoop was often too small to collect all of the feces or for the child to aim accurately, resulting in a portion of the feces still being deposited onto the ground and requiring an additional material to push the rest of the feces onto the scoop for disposal.

#### 3.4.3 Feces disposal

Multiple caregivers threw feces into an open field, which also appeared to be used for dispersed disposal of household garbage. However, when feces were disposed of in an open field, this was typically thrown in an area behind the house in a nearby area less than a 30 s walk away. At least two households disposed of children's feces by throwing them within an estimated few meters of the house where other garbage was scattered on the ground. Additionally, if feces were not removed immediately or if feces residue was visible after removal, flies quickly (<1 min) came into contact with it.

## 4 Discussion

Using mixed methods, we described practices along the entire CFM pathway, including defecation, feces handling and disposal, defecation area or tool cleaning, anal cleansing, and handwashing, and identified how each of these practices can influence potential fecal contamination of the environment and hands. Additionally, *E. coli* results from samples collected using a paired sample collection strategy at the time of defecation and feces disposal provided empirical evidence that defecating on the floor or ground increased the level of contamination on the floor after feces were removed, even if paper was laid down prior to defecation. These results also provided experimental evidence for classifying certain feces handling practices that increased contamination on caregiver's hands as unsafe (paper, a plastic bag, or straw/hay) and other practices that did not increase contamination as safe (potty, scoop, or hoe). Overall, this study expands evidence related to fecal contamination associated with various CFM practices, and adds to the growing body of evidence (Bauza et al., 2019a; Majorin et al., 2017; Miller-Petrie et al., 2016) about the need for CFM to take a holistic approach that goes beyond feces disposal to address additional steps along the CFM pathway.

Several practices were identified that lead to fecal contamination of the environment during child defecation. Defecating on the ground or finished floor was common even among children old enough to use latrines. This result is consistent with other studies in India and Bangladesh which have also reported the ground (inside or outside the household) to be the most common defecation location for young children (Bauza et al., 2019b; George et al., 2016; Majorin et al., 2014, Majorin et al., 2017). However, sample analysis provided evidence that defecating on the floor or ground increased the level of contamination on the floor or ground after feces were removed, even if paper was laid down prior to defecation. These defecation areas were also commonly reported to be cleaned with water (but without soap or disinfectant), which could spread the contamination left on the floor or ground rather than eliminating this contamination. Further, children were observed to defecate in various places in and around the household indicating that the contamination may not be confined, but widespread. When defecating onto or into other items, such as a potty, scoop, or nappy/clothing, some households reported cleaning these in or near the compound or in surface water where feces residue remaining on these tools could contaminate the environment during cleaning, and the high *E. coli* levels measured on the scoops and potties in this study provides evidence that fecal contamination remains on tools after feces disposal and initial cleaning. Other studies have also reported that water from washing cloth diapers (Yeager et al., 1999) or potties (Bauza et al., 2019a) may be dumped close to the home. Additionally, a recent study in Kenya found that fecal contamination from young children was pervasive in the domestic environment both inside and outside the household, with young children's feces being a more common source of human fecal contamination in households than older children/adult's feces (Bauza et al., 2019a), and our study supports this finding by providing evidence of specific CFM practices that could lead to contamination from young children's feces inside households.

Further down the CFM pathway, feces disposal practices and anal cleansing practices were also identified that were likely to contaminate the environment and/or caregivers hands. When caregivers picked up children's feces to dispose of it, waste newspaper/paper was most commonly used for this. Sampling revealed that use of these unsafe materials, such as paper, cloth, or straw/hay increased the level of contamination on hands, whereas the use of safe materials such as a potty, hoe, or scoop did not increase hand contamination. Throwing feces into an open field was also the most common disposal location for children <6 years and unstructured observation revealed that this field was usually close to the household which could potentially lead to contamination exposure from contact with flies, animals, or be spread during rain. Anal cleansing of children was most commonly practiced using water and in a location that could contaminate the environment (e.g., at the handpump, on the household floor, on the ground in the compound) for the majority of children <6 years. Although anal cleansing methods are rarely reported, our findings related to unsafe child feces disposal are consistent with past studies in rural Bangladesh and India. Disposal in an open space adjacent to the household compound was the most common disposal location for children's feces in a study of 216 Bangladeshi children aged 6–30 months (George et al., 2016) whereas disposal in the household's solid waste disposal site that was usually located outside at the rear of the household compound was the most common disposal location in a study of 145 children under 5 years in Odisha, India (Majorin et al., 2014).

CFM hardware that creates a better barrier between feces and the environment or hands than the commonly used materials of paper or straw/hay could be beneficial for reducing or eliminating fecal contamination exposure. The CFM hardware distributed as part of an intervention within the study area was similar to hardware typically identified or promoted for CFM (e.g., diapers/nappies, scoops/hoes, potties) (Luby et al., 2018; Miller-Petrie et al., 2016; Null et al., 2018). Although there was evidence that the CFM hardware (potties and scoops) in the study area reduced fecal contamination when used, our results also suggest that their use was not optimal and that the hardware had shortfalls. Results from a previous assessment of potties in 26 households with children <3 years in Bangladesh found that while younger children were successful at using potties, children 13–24 months of age would typically sit on a potty but resist defecating in it and children over 2 years would typically refuse to sit on a potty to defecate (Hussain et al., 2017). Caregivers in Bangladesh believed that these older children did not want to use the potties because they had already developed the habit of defecating in the open (Hussain et al., 2017), which could have also been one reason we saw low use of potties in this study. Another reason may be the design of potties; as the normal position for defecating in this population is squatting rather than sitting, an alternative to potties that is suitable to squatting during defecation may be an easier intervention for children to use and it may be a more natural progression of their defecation behavior.

Future studies should explore and evaluate CFM hardware that can be used while squatting as well as hardware and behavior messaging to encourage earlier initiation of latrine training. CFM hardware adaptations, such as a latrine training mat that can be placed over the latrine hole/pan to make it smaller (Van Schoyck, 2012) or a squatting-based potty, may be useful for increasing safe disposal in this population where it is the norm to squat during defecation. There is also potential for earlier initiation of latrine training among children, as the median age reported for beginning latrine training was 4 years and children may be developmentally ready to squat over a latrine at a younger age. Some caregivers reported the potential for children to fall in the latrine squatting pan or pit as a reason children 3 and 4 years of age should not use the latrine. This also suggests that an intervention to decrease the latrine squatting pan hole size and/or provide a handrail/stable item for a child to hold while defecating could make the latrine more user-friendly for children and potentially increase child latrine use by mitigating this safety concern. For younger children not yet ready to use a latrine, a larger scoop that children could completely squat over may be a more effective scoop design that could prevent the need for a child to defecate on the floor/ground and the use of additional disposal items to transfer feces from floor/ground to the scoop, both of which could introduce additional contamination into the environment. New CFM hardware co-designed with the community could also potentially improve functionality and use by better accounting for local customs and practices.

Our sampling strategy provided an evidence-based method of classifying certain CFM practices as safe or unsafe and could be useful for a greater range of CFM practices in future studies. The sample strategy involved collecting paired samples at the time and place of defecation and feces disposal to get more targeted activity-level evidence of contamination related to specific CFM activities. The paired samples included a sample for the targeted activity as well as a similar sample to measure existing background contamination of *E. coli* in the environment or on hands. This type of activity-level sampling which accounts for background contamination could be useful for evaluating CFM practices, and may provide more information than typical sampling in WASH studies which is completed by collecting grab samples around the domestic environment at the time of a household visit. Grab samples from drinking water, hand rinses, or other domestic locations at a single time-point may be convenient to collect, but may not be an effective way to measure reductions in fecal-oral pathways as *E. coli* can be naturally occurring in the environment and may not correlate with fecal-oral disease outcomes following interventions (Ercumen et al., 2018b).

We also found a high level of variability between households related to fecal contamination resulting from CFM practices, including high variability in the level of contamination measured for the same CFM practices in different households. Some variability in how the same types of practices are performed in different households was observed during unstructured observation. In particular, different types and thicknesses of paper were often used by different households, which could affect the level of fecal contamination that penetrates the paper to contaminate the ground or floor underneath or the caregivers hands that are picking up and transporting the feces to the disposal location, but this type of information is not typically reported during household surveys. Additionally, whether the ground/floor or paper was wet may have impacted the level of fecal contamination that was transferred from the feces to the ground or caregiver's hands. Due to the potential for high variability in fecal contamination, interventions for safe CFM may require the inclusion of detailed messaging on the specific way that CFM practices should be performed, including the correct and incorrect usage of hardware.

This study had several limitations. First, there were small sample sizes for many of the practices measured, including safe practices, which limits the comparisons we can make and conclusions that we can draw from the data. We selected households to include in the study that had received potties and scoops as part of an intervention to collect samples for safe and unsafe practices, however, fewer households were using the CFM hardware and safe practices than we expected. Additionally, although we tried to assess the potential for fecal contamination from all steps along the CFM pathway, we were unable to collect and analyze samples for all of these practices. In particular, we were planning to collect samples surrounding anal cleansing when it was completed in the household or compound, but this turned out to not be feasible and should be investigated in future studies. Future studies could also consider sampling to better quantify contamination introduced from cleaning materials like nappies or scoops using only water. Additionally, we did not assess child hand contamination or child handwashing after defecation. This step should be assessed in future studies, as a past study in Bangladesh found low handwashing of young children following defecation (George et al., 2016). Child handwashing may be particularly important for children who are using latrines, as latrine use without proper handwashing may increase the level of contamination on children's hands (Greene et al., 2012). Furthermore, while efforts were made to sample the exact area on the ground or floor that a child defecated on by placing toothpicks around the feces prior to the feces being removed, toothpicks were instead placed around the edge of the paper when a child defecated on paper. It was still attempted to sample the area of the ground from under the paper were feces had been, but sometimes the paper was quite large (e.g., full 2-page newspapers) and the area sampled may not have been the exact area of defecation which would have reduced the difference in contamination levels that we saw between the area of defecation and background level of contamination from the area near defecation. Finally, while one study in Bangladesh found low levels of reporting bias for safe disposal behaviors (George et al., 2016), other studies have noted more caregivers reporting desirable hygienic behaviors during surveys than those who are actually observed to perform such behaviors (Curtis et al., 1993; Manun'Ebo et al., 1997), so it is possible that our surveys over-report safe CFM and handwashing behaviors.

Despite its limitations, our study demonstrated a sampling technique at the activity-level for steps along the CFM pathway, and used this technique to provide empirical evidence that current tools widely used for child defecation (such as placing paper on the ground) and picking up feces (such as using paper or straw/hay) are not effective at preventing fecal contamination of the ground/floor or caregiver hands. In combination with sample collection, we also used information collected from surveys and unstructured observations to capture information on the likely fecal contamination occurring from all practices related to CFM, including defecation, feces handling and disposal, defecation area and/or tool cleaning, anal cleansing, and handwashing. We identified shortcomings with the CFM hardware interventions of scoops and potties in this study area and made recommendations to improve future CFM hardware interventions. In particular, CFM interventions that initiate latrine training at an earlier age or can safely collect feces while a child remains squatting during defecation may be particularly beneficial, such as hardware to reduce the size of the hole opening in a latrine to make a latrine more child-friendly. Further research is also needed to design and evaluate new CFM hardware interventions, particularly ones that comprehensively account for defecation, feces handling and disposal, and cleaning activities to reduce contamination in the environment and on caregivers' hands. Finally, as fecal contamination can originate from many different steps along the CFM pathway, there is a need for the WASH field to move beyond only assessing the final disposal location of child feces, and future studies and programs should focus on safe CFM as a holistic set of practices with safe practices considered during defecation, feces handling, feces disposal, cleaning of CFM tools, anal cleansing, and handwashing.

## Declaration of competing interest

The authors declare that they have no known competing financial interests or personal relationships that could have appeared to influence the work reported in this paper.

## Appendix A Supplementary data

Supplementary material Image 1
