# Supplementary material for: Child feces management practices and fecal contamination: A cross-sectional study in rural Odisha, India
Source: Sci Total Environ. 2020 Mar 20;709:136169. doi: 10.1016/j.scitotenv.2019.136169 (PMC7031693; doi:10.1016/j.scitotenv.2019.136169)
Supplement: Supplementary file 1 — Supplementary material [file mmc1.pdf]

SUPPLEMENTAL INFORMATION

Child feces management practices and fecal contamination: a cross-sectional study in rural Odisha, India

Valerie Bauza,[1*] Fiona Majorin,[2] Parimita Routray,[3] Gloria Sclar,[1] Bethany Caruso,[1] Thomas Clasen[1]

[1] Department of Environmental Health, Rollins School of Public Health, Emory University, Atlanta, Georgia, United States of America
[2] London School of Hygiene and Tropical Medicine, London, United Kingdom
[3] Independent Consultant, Bhubaneswar, Odisha, India

*Corresponding author email: valerie.bauza@emory.edu

Figures

Figure S1: Pictures of the potty and scoop distributed to intervention villages
Figure S1: Pictures of the variations in latrine types in study villages

Tables

Table S1: Defecation location reported for the last time each household member defecated, by gender
Table S2: Caregiver’s perceptions of child ages appropriate for potty and latrine use.
Table S3: Defecation location reported for the last time each household member defecated, separated by households in intervention and control villages.
Table S4: Child feces disposal locations for the last time the child defecated, separated by households in intervention and control villages.

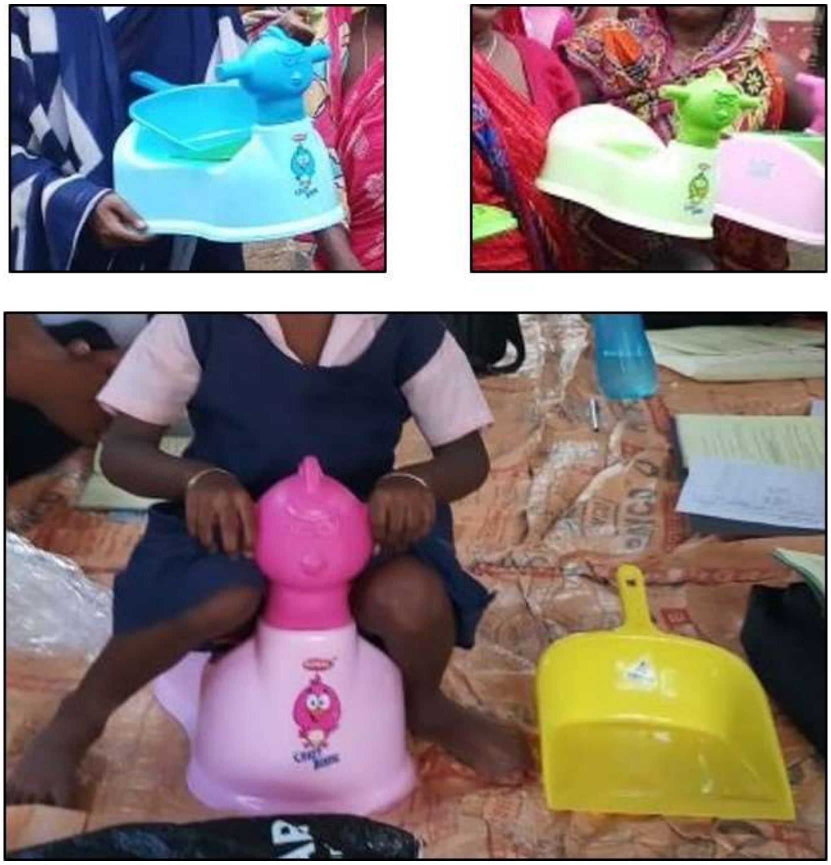

Figure S1. Pictures of the potty and scoop distributed to intervention villages

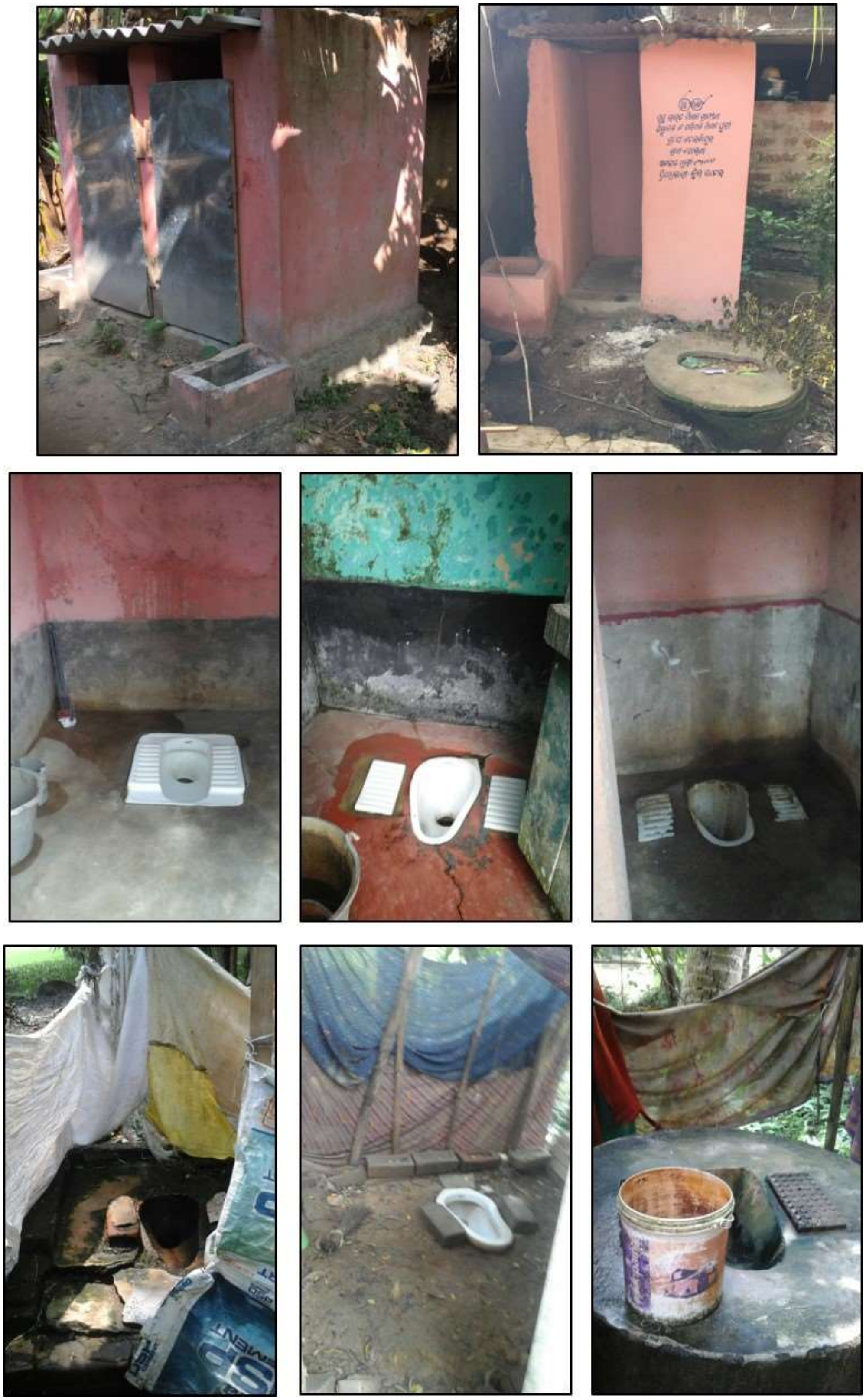

Figure S2. Pictures of the variations in latrine types in study villages

Table S1. Defecation location reported for the last time each household member defecated, by gender.

	<3 yr	3-5 yr	6-9 yr	10-14 yr	15-59 yr	60+ yr	All
Female							
N	54	52	45	26	308	59	544
In latrine	3.7%	34.6%	71.1%	80.8	78.3%	67.8%	65.1%
On ground outside compound	3.7%	13.5%	17.8%	15.4%	18.8%	30.5%	17.8%
On ground inside compound	31.5%	36.5%	11.1%	-	-	-	7.5%
On floor in house	33.3%	9.6%	-	-	-	-	4.2%
In potty	7.4%	1.9%	-	-	0.3%	-	1.1%
In cloth nappy/diaper	1.9%	-	-	-	-	-	0.2%
In pants/clothing	1.9%	1.9%	-	-	-	-	0.4%
On bed	13.0%	-	-	-	-	-	1.3%
In bedpan	-	-	-	-	-	1.7%	0.2%
Other	-	-	-	-	-	-	-
Don't know	3.7%	1.9%	-	3.9%	2.6%	-	2.2%
Male							
N	48	72	39	10	278	81	528
In latrine	4.2%	38.9%	69.2%	80.0%	64.0%	67.9%	56.5%
On ground outside compound	8.3%	9.7%	23.1%	10.0%	27.7%	30.9%	23.3%
On ground inside compound	27.1%	43.1%	7.7%	10.0%	0.7%	-	9.5%
On floor in house	41.7%	1.4%	-	-	-	-	4.0%
In potty	4.2%	2.8%	-	-	-	-	0.8%
In cloth nappy/diaper	2.1%	-	-	-	-	-	0.2%
In pants/clothing	4.2%	-	-	-	-	-	0.4%
On bed	6.3%	-	-	-	-	-	0.6%
In bedpan	-	-	-	-	0.4%	-	0.2%
Other	-	2.8%	-	-	-	-	0.2%
Don't know	2.1%	1.4%	-	-	7.2%	1.2%	4.4%

Table S2. Caregiver's perceptions of child ages appropriate for potty and latrine use.

Age (years)	Potty use (N=81)		Latrine use (N=188)	
	Appropriate age for a child to begin using a potty?	Age a child is too old to use a potty?	Age to begin training your child to use a latrine?	Age at which your child should be able to use a latrine on their own without assistance?
<1	1.2%	-	-	-
1	50.6%	1.2%	1.1%	-
2	27.2%	17.3%	13.8%	-
3	2.5%	28.4%	26.1%	1.6%
4	1.2%	13.6%	26.1%	5.9%
5	-	19.8%	21.8%	21.3%
6	-	2.5%	3.7%	25.5%
7	-	-	0.5%	15.4%
8	-	-	-	18.1%
9	-	-	-	1.6%
10	-	-	-	3.7%
Don't know	17.3%	17.3%	6.9%	6.9%

Table S3. Defecation location reported for the last time each household member defecated, separated by households in intervention and control villages.

	<3 years	3-5 yr	6-9 yr	10-14 yr	15-59 yr	60+ yr
Intervention villages						
N	50	61	40	17	298	72
In latrine	4.0%	46.8%	70.0%	94.1%	77.5%	66.7%
On ground outside compound	4.0%	11.3%	20.0%	5.9%	17.8%	31.9%
On ground inside compound	28.0%	29.0%	10.0%	-	-	-
On floor in house	34.0%	1.6%	-	-	-	-
In potty	10.0%	4.8%	-	-	0.3%	-
In cloth nappy/diaper	2.0%	-	-	-	-	-
In pants/clothing	2.0%	1.6%	-	-	-	-
On bed	12.0%	-	-	-	-	-
In bedpan	-	-	-	-	-	-
Other	-	3.2%	-	-	-	-
Don't know	4.0%	1.6%	-	-	4.4%	1.4%
Control villages						
N	52	62	44	19	288	68
In latrine	3.9%	27.4%	70.5%	68.4%	65.3%	69.1%
On ground outside compound	7.7%	11.3%	20.5%	21.1%	28.5%	29.4%
On ground inside compound	30.8%	51.6%	9.1%	5.3%	0.7%	-
On floor in house	40.4%	8.1%	-	-	-	-
In potty	1.9%	-	-	-	-	-
In cloth nappy/diaper	1.9%	-	-	-	-	-
In pants/clothing	3.9%	-	-	-	-	-
On bed	7.7%	-	-	-	-	-
In bedpan	-	-	-	-	0.4%	1.5%
Other	-	-	-	-	-	-
Don't know	1.9%	1.6%	-	5.3%	5.2%	-

Table S4. Child feces disposal locations for the last time the child defecated, separated by households in intervention and control villages.

	<3 years	3-5 yr	6-9 yr	All <10 yr
Intervention villages				
N	50	62	40	152
Child used latrine	4.0%	46.8%	70.0%	38.8%
Put/rinsed in latrine	26.0%	9.7%	-	12.5%
Put/rinsed in drain/ditch	2.0%	3.2%	-	2.0%
Thrown in garbage	4.0%	4.8%	2.5%	4.0%
Thrown in open field	36.0%	24.2%	10.0%	24.3%
Left in open	-	8.1%	17.5%	7.9%
Put/rinsed in pond/surface water	14.0%	1.6%	-	5.3%
Washed away with soap and water	10.0%	-	-	3.3%
Don’t know	4.0%	1.6%	-	2.0%
Control villages				
N	52	62	44	158
Child used latrine	3.9%	25.8%	70.5%	31.0%
Put/rinsed in latrine	7.7%	1.6%	-	3.2%
Put/rinsed in drain/ditch	-	-	-	-
Thrown in garbage	19.2%	11.3%	2.3%	11.4%
Thrown in open field	50.0%	56.5%	9.1%	41.1%
Left in open	-	3.2%	18.2%	6.3%
Put/rinsed in pond/surface water	15.4%	-	-	5.1%
Washed away with soap and water	1.9%	-	-	0.6%
Don’t know	1.9%	1.6%	-	1.3%
